# Gene-centromere mapping in meiotic gynogenetic European seabass

**DOI:** 10.1186/s12864-017-3826-z

**Published:** 2017-06-07

**Authors:** Münevver Oral, Julie Colléter, Michaël Bekaert, John B Taggart, Christos Palaiokostas, Brendan J. McAndrew, Marc Vandeputte, Béatrice Chatain, Heiner Kuhl, Richard Reinhardt, Stefano Peruzzi, David J Penman

**Affiliations:** 1 Institute of Aquaculture, School of Natural Sciences, University of Stirling, FK9 4LA Stirling, Scotland UK; 2 Cirad, Persyst, UMR Intrepid, Campus International de Baillarguet, 34398 Montpellier, France; 3 Ifremer, 34250 Palavas-Les-Flots, France; 4 INRA, GABI, AgroParisTech, Université Paris-Saclay, 78350 Jouy-en-Josas, France; 5 Leibniz-Institute of Freshwater Biology and Inland Fisheries, Müggelseedamm 310, 12587 Berlin, Germany; 6 Max-Planck-Institute for Plant Breeding, Max-Planck Genome Centre Cologne, Carl-von-LinnÃ©-Weg 10, D-50829 Cologne, Germany; 7 Department of Arctic and Marine Biology, Faculty of Biosciences, Fisheries and Economics, University of Tromsø, 9037 Breivika Tromsø, Norway

**Keywords:** *Dicentrarchus labrax*, Meiotic gynogenesis, Isogenic lines, ddRAD seq, Genetic map, Gene-Centromere map, Aquaculture

## Abstract

**Background:**

Fully isogenic lines in fish can be developed using “mitotic” gynogenesis (suppression of first zygotic mitosis following inactivation of the sperm genome). However, genome-wide verification of the steps in this process has seldom been applied. We used ddRADseq to generate SNP markers in a meiotic gynogenetic family of European seabass (*Dicentrarchus labrax*): (i) to verify the lack of paternal contribution in a meiotic gynogenetic family; (ii) to generate a gene-centromere map from this family; (iii) to identify telomeric markers that could distinguish mitotic gynogenetics from meiotic gynogenetics, which sometimes arise spontaneously in mitotic gynogenetic families.

**Results:**

From a single meiotic gynogenetic family consisting of 79 progeny, 42 million sequencing reads (Illumina, trimmed to 148 bases) resolved 6866 unique RAD-tags. The 340 male-informative SNP markers that were identified confirmed the lack of paternal contribution. A gene-centromere map was constructed based on 804 female-informative SNPs in 24 linkage groups (2n = 48) with a total length of 1251.02 cM (initial LG assignment was based on the seabass genome assembly, dicLab v1). Chromosome arm structure could be clearly discerned from the pattern of heterozygosity in each linkage group in 18 out of 24 LGs: the other six showed anomalies that appeared to be related to issues in the genome assembly.

**Conclusion:**

Genome-wide screening enabled substantive verification of the production of the gynogenetic family used in this study. The large number of telomeric and subtelomeric markers with high heterozygosity values in the meiotic gynogenetic family indicate that such markers could be used to clearly distinguish between meiotic and mitotic gynogenetics.

**Electronic supplementary material:**

The online version of this article (doi:10.1186/s12864-017-3826-z) contains supplementary material, which is available to authorized users.

## Background

Polyploidy has occurred during evolution of various fish groups [1] while gynogenesis is a natural form of reproduction in some species [2]. Spontaneous polyploids have also been observed in both wild and farmed fish [1]. Induced chromosome set manipulation is a methodology that has been exploited over a long period in fish research [3–7]. The ability to retain the second polar body post-fertilisation and/or suppress first cell division by temperature, chemical or pressure shocks, coupled with the relative ease of gamete inactivation by irradiation has led to its widespread use. The various chromosome sets that can be generated (haploids, triploids, tetraploids, androgenetics, meiotic or mitotic gynogenetics) have been exploited in a wide range of studies including gene mapping [8, 9], genome assembly [10, 11], construction of isogenic lines [12, 13] and production of sterile farm fish [14, 15].

European seabass is an important mariculture species, extensively farmed in the Mediterranean basin. The need to develop genetic and genomic resources to underpin future development of this species is clearly recognised, and has resulted in the production of a first draft genome assembly [16], a number of linkage maps [17–19] and a radiation hybrid panel [20]. A further key resource would be the development of isogenic lines through androgenesis [21] or mitotic gynogenesis [22]. These have not been successfully established yet, despite significant efforts [21–24].

Though widely practised, there are a number of technical pitfalls that can impact the effectiveness of induced gynogenesis and androgenesis. For example, there can be a potential genetic contribution from the irradiated gamete source, this being associated with poorly optimised protocols leading to incomplete inactivation [2]. Furthermore, the efficiency of protocols designed to retain chromosome sets post fertilisation/activation can also be severely affected by gamete quality and slight alterations in the timing and intensity of the applied shock [22, 25, 26]. Spontaneous retention of the second polar body [27, 28] may also generate additional unexpected (and unwanted) ploidy states. One of the bottlenecks in production of isogenic lines through mitotic gynogenesis is spontaneous meiotic gynogenetics [29], which have some level of heterozygosity through retention of the second polar body, and need to be detected and eliminated from putative mitotic gynogenetic fish for the reliable production of isogenic lines in the subsequent generation.

Throughout the development of the technology, genetic markers have been used to monitor the effectiveness of the procedure. To date this has generally involved screening with a small panel of available markers, to confirm the presence/absence of particular parental chromosomal sets. These markers include pigmentation genes, allozymes, multilocus minisatellites and microsatellites [2]. While this approach can give an indication as to the effectiveness of the treatment, it is relatively insensitive for detection and quantification of potential instances of aneuploidy. Another limitation to using a small number of markers is that those that happen to be located close to centromeric regions will be compromised with respect to their ability to detect crossover events. This is a key requirement, for example, for differentiating between mitotic and meiotic gynogenetics; i.e. informative (heterozygous in the dam) telomeric markers will be heterozygous in meiotic gynogenetics and homozygous in mitotic gynogenetics, while informative centromeric markers will largely be homozygous in both types.

The advent of genotyping by sequencing approaches that exploit next generation sequencing technologies [30] permits the simultaneous discovery and screening of large numbers of single nucleotide polymorphisms (SNPs) per individual at a realistic cost. This provides an opportunity to more accurately assess the effectiveness of various elements of chromosomal set manipulation procedures. In this study SNPs generated by double digest restriction associated DNA (ddRAD) sequencing (ddRAD seq; [31]) were employed to comprehensively examine parental genetic contributions in an experimentally generated meiotic gynogenetic family of European seabass, *Dicentrarchus labrax.* The main objectives of the study were to (i) look for potential paternal contribution from UV-irradiated sperm; (ii) generate a SNP locus - centromere map; and iii) screen informative (female heterozygous) markers for their potential to distinguish between mitotic and meiotic gynogenetics.

## Methods

### Production of mapping family – Meiotic gynogenetics

The meiotic gynogenetic seabass family was produced at the Ifremer Experimental Aquaculture Station (Palavas-les-Flots, France), using parent fish from a West Mediterranean broodstock population. Broodstock were aged 4 to 6 years and weighed 1 to 5 kg, and were kept in recirculating systems (8 m^3^ tanks, rate of O_2_ enriched water renewal 250 Lh^−1^, constant low aeration) maintained under natural conditions of temperature and photoperiod (43° 31′ 40 N, 3° 55′ 37 E) and fed commercial diets (NeoRepro, Le Gouessant, France). Spermiating males were identified by gentle abdominal pressure and held in a handling tank. Female maturation stage was assessed from ovarian biopsies obtained by introducing a thin catheter (Pipelle de Cornier, Laboratoire CCD, Paris, France) into the genital orifice. Females at the correct stage of development received a single dose (10 μg.kg^−1^) of Luteinizing Hormone Releasing Hormone analogue (LHRHa, Sigma, France) in order to induce final maturation and ovulation. The UV irradiation device, used to inactivate the paternal genome, comprised of eight UV lamps (12 W, 254 nm, Vilber-Lourmat, Marne-la-Vallée, France) fixed above and below (four lamps each) a quartz plate which was mechanically agitated to stir sperm samples throughout irradiation. Diluted sperm (5 mL) from a single male (diluted 1:20, *v*/v in artificial extender SGSS [Seabass Gamete Short term Storage] made of StorFish [IMV Technologies, France] complemented with pyruvate and glutamine at 0.6 and 3 mg/ml^−1^ respectively [C. Fauvel, pers. comm.]) was irradiated in an 8.5 cm diameter quartz petri dish for 8 min to apply a total dose of 326 mJ/cm^2^ [23].

The irradiated sperm were added to 125 mL of eggs (untreated, good quality) collected from a single female and then 125 mL of seawater was added to initiate fertilisation. A pressure shock of 8500 psi and 2 min duration was applied, starting at 6 min after fertilisation, to restore diploidy via retention of the second polar body [23]. All procedures were performed under total darkness in a temperature-controlled room maintained at 14 °C. Eggs were incubated in 40 L tanks in a dedicated recirculating water system (temperature 14–14.5 °C; salinity 35–36‰) until hatching. All tanks were maintained in darkness until sampling. Ten days after hatching, a subset of 80 larvae were fixed in 99% ethanol; fin tissue from parents was also fixed in ethanol.

### DNA preparation

DNA was extracted from all 80 offspring (entire larva) and both parents (fin tissue) using a commercial salting out kit (REALpure DNA extraction kit; REAL Laboratories, Durviz, Spain) according to the manufacturer’s protocol. This included the recommended RNase incubation step to reduce RNA contamination in the final product. The DNA concentration and purity of each sample was assessed by spectrophotometry (Nanodrop, Thermo Scientific, UK), while its integrity was assessed by 0.7% agarose gel electrophoresis. Each sample was then preliminarily diluted to c. 50 ng/μL in 5 mM Tris, pH 8.5. A final, more accurate, fluorometric-based assessment of DNA concentration was then performed on all samples using the Qubit® dsDNA HS Assay Kit (Invitrogen, UK). Fluorescence measurements (20 μL volumes) were performed on a 96 well qPCR thermal cycler (Quantica, Techne, UK), with seabass DNA concentrations being derived from a calibration curve generated from a set of standard dsDNAs. Based on these readings the seabass samples were diluted to c. 10 ng/μL in 5 mM Tris, pH 8.5 for use in ddRAD library construction protocol.

### ddRAD library preparation and sequencing

The ddRAD library preparation protocol used here is described in detail elsewhere [32, 33]. In silico estimation from the seabass genome predicted 52,230 ddRAD fragments with paired *SbfI-SphI* restriction site overhangs, while after the size selection applied in the present study (c. 320 bp −590 bp excluding adaptors) only 3603 fragments were predicted to be available.

Briefly, a single restriction enzyme digestion/adapter ligation reaction was performed for each progeny sample, while triplicate reactions were made for both dam and sire DNA samples. The latter ensured higher coverage of parental samples, which allowed more robust assignment of true SNPs in the pedigree. Each sample (40 ng DNA) was digested at 37 °C for 30 min with 0.8 U *Sbf*I (‘rare’ cutter, CCTGCA|GG motif) and 0.8 U *SphI* (‘common’ cutter, GCATG|C motif) high fidelity restriction enzymes (New England Biolabs; NEB) in a 6 μL reaction volume that included 1× CutSmart™ buffer (NEB). After cooling the reactions to room temperature, 3 μL of a premade barcode-adapter mix was added to the digested DNA, and incubated at room temperature for 10 min. This adapter mix comprised individual-specific barcoded combinations of P1 (*Sbf*I-compatible) and P2 (*Sph*I-compatible) adapters at 6 nM and 72 nM concentrations respectively, in 1× reaction buffer 2 (NEB). Adapters were compatible with Illumina sequencing chemistry (see [31] for details). The barcoded adapters were designed such that adapter–genomic DNA ligations did not reconstitute RE sites, while residual RE activity limited concatemerization of genomic fragments during ligation. The adapters included an inline five- or seven-base barcode for sample identification (Additional file 1: Table S1). Ligation was performed over 40 min at 22 °C by addition of a further 3 μL of a ligation mix comprising 4 mM rATP (Promega, UK), and 2000 cohesive-end units of T4 ligase (NEB) in 1× CutSmart buffer.

The ligated samples were then heat denatured at 65 °C for 20 min, cooled, and combined into a single pool. The pooled sample was column-purified (MinElute PCR Purification Kit, Qiagen, UK) and size selection of fragments, c. 320 bp to 590 bp, was performed by agarose gel electrophoresis. Following gel purification (MinElute Gel Extraction Kit, Qiagen, UK) the eluted size-selected template DNA (60 μL in EB buffer) was PCR amplified (11 cycles PCR; 28 separate 12.5 μL reactions, each with 1 μL template DNA) using a high fidelity Taq polymerase (Q5 Hot Start High-Fidelity DNA Polymerase, NEB). The PCR reactions were combined (350 μL total), and column-purified (MinElute PCR Purification Kit). The 55 μL elute, in EB buffer, was then subjected to a further size-selection clean up using an equal volume of AMPure magnetic beads (Perkin-Elmer, UK), to maximize removal of small fragments (less than ca. 200 bp).

The final library was eluted in 20 μL EB buffer and sequenced over two full Illumina MiSeq runs (v2 chemistry, 300 cycle kit, 162 bp paired end reads; Illumina, Cambridge, UK; 10.5 pM library applied and both runs spiked with 3% Illumina phiX control DNA). The raw sequence data from this study were deposited at the EBI Sequence Read Archive (SRA) with the accession number ERP006697.

### Genotyping ddRAD alleles

Following initial analysis (FastQC: [34]) to confirm that high-quality sequence data had been generated, the MiSeq reads were processed using Stacks (v.1. 17; [35]), a package designed specifically to identify and score SNPs from restriction-enzyme based sequence data. First, the ‘process_radtags’ function was used to demultiplex the individual samples. During this process sequence reads with quality scores below 20 (−s set to 20), missing either restriction site or with ambiguous barcodes were discarded. Barcodes were removed and all sequences were 3′ end trimmed to be 148 bases long. Then reference based Stacks analysis was performed, using ‘ref_map.pl’ perl script. Sequence alignment/map (SAM) files were created using Bowtie 2 aligner [36] and the seabass genome (dicLab v1; [16]). The main Stacks parameter values used in this analysis were m = 10 and *n* = 1. In order to maximise the number of informative markers investigated while minimising missing or erroneous data, only polymorphic ddRAD-tags that containing 3 or less SNPs (maximum of 4 alleles) and which were detected in both parents and present in at least 75% of the offspring were scored.

### Genetic linkage map construction

It was not feasible to construct a genetic linkage map de novo from the unordered meiotic gynogenetic family data. Both R/OneMap [37] and TMAP [38] were explored for genetic linkage map construction without success. The final map was constructed using R/OneMap after assigning markers to linkage groups based on the seabass genome assembly (see section *h* below). Genotypes were imported in *outcross* format into R/OneMap in a modified way such that all genotypes shared the same segregation pattern (“ab x ab cross”). This package uses Hidden Markov Models (HMM) algorithms for outbred species while in parallel implements the methodology described in [39], for calculating the most probable linkage phase. Recombination fraction between all pairs of markers was calculated using *rf.2pts* function. These groups were ordered using the *order.seq* function in four available two-point based algorithms including *ser*, *rcd*, *rec* and μg and the one which gave the smallest distance was selected for each LG. Following ordering, markers in the same LG were forced to the final map by using *force* function after inspection of *safe* order. The order of markers was also inspected visually using *rf.graph.table* which plots a heat map of LOD score and recombination frequency. Map distances were calculated in centiMorgans (cM) using the Kosambi mapping function. Genetic Mapper v0.5 [40] was used for the final visualisation of genetic linkage map of meiotic gynogenetic *D.labrax*.

### Visualising physical position of markers and microsatellites from previous studies

Outputs of genome aligner (SAM files) were used for the positioning each ddRADseq locus and visualised using Genetic Mapper v0.5 [40]. Eleven microsatellite markers [17, 41] that have been used to differentiate between meiotic and mitotic gynogenetic sea bass [29] were also assigned to the physical map once the genomic positions were identified using Blastn (10^−20^ and lower).

### Marker-centromere mapping

Centromeres are expected to be in regions with zero or low heterozygote frequency, with an increase in heterozygote frequency towards the telomeres. For each maternally informative ddRADseq locus, heterozygosity (*y*) was computed across all progeny. Marker-centromere map distances (in cM) were calculated using the formula 100*(*y*/2), under the assumption of complete interference, believed to be characteristic of fish species [4, 42, 43].

### Comparison of genomic assembly with linkage maps

The genome assembly and the linkage map generated in the present study were compared to the recently published RAD-based high-density SNP-based genetic linkage map of Palaiokostas et al. [19], as an independent source for comparing marker order. Common polymorphic loci between the two linkage maps were identified by BLASTn. First, the loci beginning with the common enzyme recognition site motif (“TGCAGG”; *Sbf*I) from the present study (in total: 395 markers out of 764 female heterogametic assigned markers) were trimmed down to 95 bp, compatible with the RADseq P1 read length of [19]. Then a local nucleotide database was generated on Bioedit (version 7.2.5: [44]) from all assigned markers of [19] and all polymorphic markers of the present study were blasted against them. Stringent filtering options were applied to tabular output based on: i) e value ≤ 10^−20^, and ii) alignment length ≥ 90 bases (i.e. at least 94.7% similarity).

### Estimation of recombination frequency per chromosome arm

Scoring of homozygote/heterozygote distribution along the LGs of each individual progeny was used to estimate recombination, where a change from a region of homozygous markers (defined as at least two consecutive markers with the same status) to a region of heterozygous markers was taken as a crossover point. This analysis was carried out on 18 chromosomes where chromosome arm structure could be discerned (see Results).

## Results

### ddRAD sequencing

**Fig. 1:**
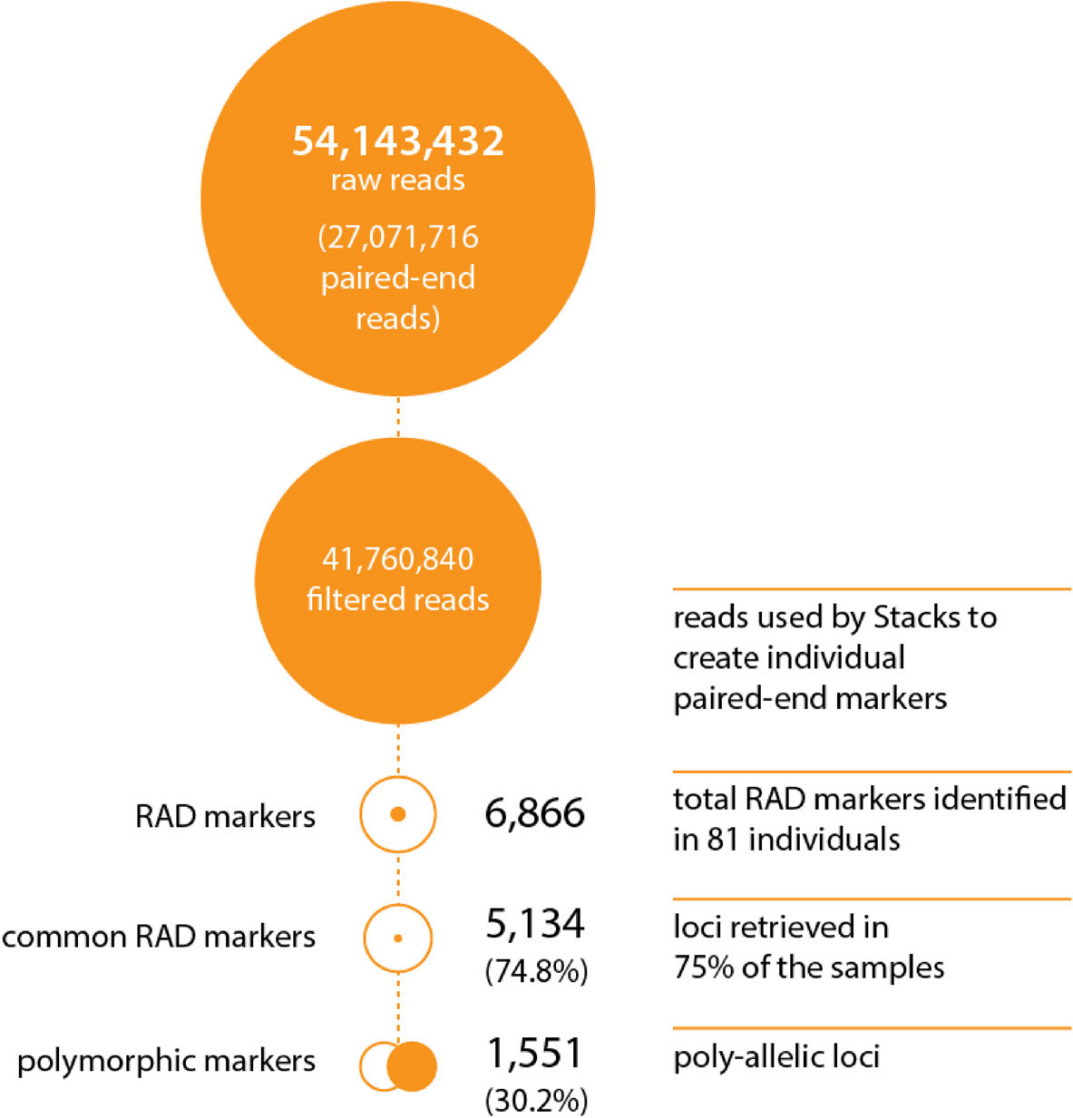
Sequencing and ddRAD-tag summary. Detailed number of reads before and the after filters (orange disk) followed by the reconstructed numbers of ddRAD markers and polymorphic ddRAD markers (orange circles)

A total of 27,071,716 paired-end raw reads were produced from the combined two sequencing runs for the meiotic gynogenetic *D. labrax* family with 79 progeny (Additional file 1: Table S1). Following demultiplexing using *process_radtags*, 77.1% of the raw paired-end reads were retained (20,880,420). Only one sample offspring (MO241) failed to produce sufficient reads (c.1542 reads <150 K) and was dropped from subsequent STACKS analyses. As planned, the read numbers for both parents (785 K, sire & 1127 K, dam) exceeded those of offspring by a factor of c. 2 (average no. per reads per offspring, 504 K). Read numbers for each sample are detailed in Additional file 2: Table S2. The reference-based Stacks analysis identified 6886 unique ddRAD loci and 1551 potential SNP loci (Fig. 1).

### Investigation of potential sire contribution

Within the polymorphic marker dataset, 340 SNPs were identified with male-informative alleles, i.e. one (214 loci) or both (126 loci) alleles at a locus detected in the male parent alone. No male-specific alleles were detected in any of the offspring. Later mapping of these loci to the seabass reference genome confirmed that these markers were located across all seabass chromosomes. Thus no sire contribution was detected within the ddRAD dataset for this gynogenetic family.

### Construction of female genetic linkage map

With the absence of paternal alleles confirmed, the marker dataset was refined to produce a robust set of informative SNPs for female map construction. Dam homozygous markers were removed (non-informative: 687 loci) as were loci where the minor allele frequency was <0.4 among the progeny samples (8 loci). Additionally 52 loci were removed since both parental genotypes were missing. This left data from 804 female-informative SNPs to be used in linkage map construction. The position of each SNP marker in the genome assembly is shown in Additional file 3: Table S3. The genomic position and informativeness of microsatellites used by [29] are shown in Additional file 4: Table S4, and both sets of markers are integrated into a physical map in Additional file 5: Fig. S1.

**Fig. 2:**
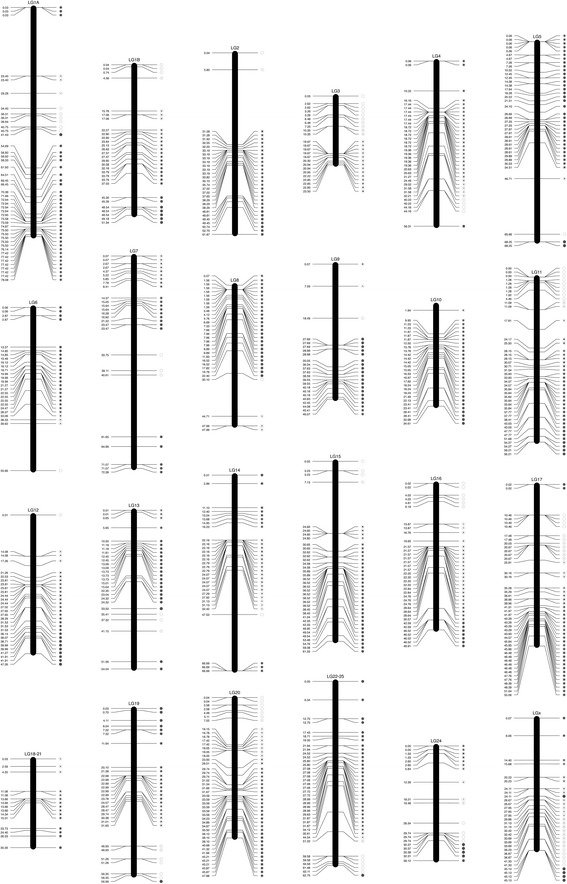
Genetic linkage map of meiotic gynogenetic *D. labrax*. The positions on the left side of chromosomes are the distance in centiMorgans (cM), the circles on the right hand side represent observed heterozygosity levels at each map position (empty circles represent homozygotes whereas increasingly filled black dots represents the higher levels of heterozygosity). Detailed data are provided in Additional file 3: Table S3

The linkage map (constructed using a LOD score of 4–5) comprised 764 SNPs and was 1251 cM in length (Fig. 2; Table 1; Additional file 3: Table S3; Additional file 6: Dataset S1). Average marker distance was 1.63 cM with 448 markers possessing unique positions. Linkage groups were between 23 cM (LG 3) and 78 cM (LG 1A) in length (mean 52 cM) and comprised between 15 (LG 18–21) and 46 markers (LG20; mean 32). As the initial grouping of SNPs within the linkage map was based on the genome assembly, the distribution of markers was in accordance with 24 chromosome pairs in *D. labrax* (originally identified from karyotype analyses; [45]).

**Table 1:** Summary of *D. labrax* genetic linkage map from a meiotic gynogenetic family, and assessment of chromosome structure

LGs	No. of markers	Size (cM)	Chromosome structure
LG 1A	45	78.04	ambiguous
LG 1B	29	51.30	mono-arm
LG 2	30	61.83	mono-arm
LG 3	24	22.79	mono-arm
LG 4	34	44.10	mono-arm
LG 5	38	68.19	ambiguous
LG 6	26	55.59	mono-arm
LG 7	26	72.31	ambiguous
LG 8	31	47.92	ambiguous
LG 9	23	46.00	ambiguous
LG 10	30	34.68	mono-arm
LG 11	42	54.26	mono-arm
LG 12	29	47.25	mono-arm
LG 13	27	54.03	ambiguous
LG 14	31	66.67	bi-arm
LG 15	37	61.31	mono-arm
LG 16	37	49.89	mono-arm
LG 17	45	55.03	bi-arm
LG 18–21	15	30.23	mono-arm
LG 19	30	56.96	mono-arm
LG 20	46	45.82	mono-arm
LG 22–25	39	62.70	mono-arm
LG 24	19	39.07	bi-arm
(LG X)	31	45.05	assembly artefact
Total	764	1251.02	

### Marker-centromere mapping

**Fig. 3:**
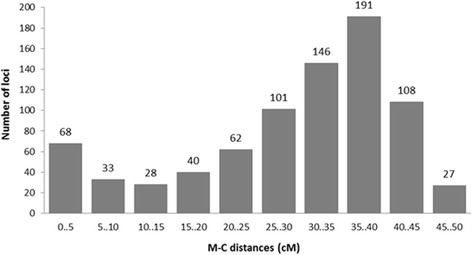
Frequency distribution of marker-centromere distances, under the assumption of complete interference at 804 female heterogametic loci in meiotic gynogenetic European Seabass

Heterozygote frequencies for 804 female informative markers in the meiotic gynogenetic family ranged between zero and one (i.e. 0 to 50 cM map distances under the assumption of complete interference). Figure 3 shows a histogram of recombination frequencies and Additional file 7: Table S5 shows marker-centromere map distances. Seven loci (0.87% of total loci) showed 100% recombination (i.e. telomeric), while 16 loci (1.99%) showed zero recombination (i.e. centromeric). Almost half of the markers had heterozygote frequencies above 0.667 (49.12%), the expected maximum theoretical value for independent segregation between a marker and the centromere when multiple crossovers occur, indicating high interference.

**Fig. 4:**
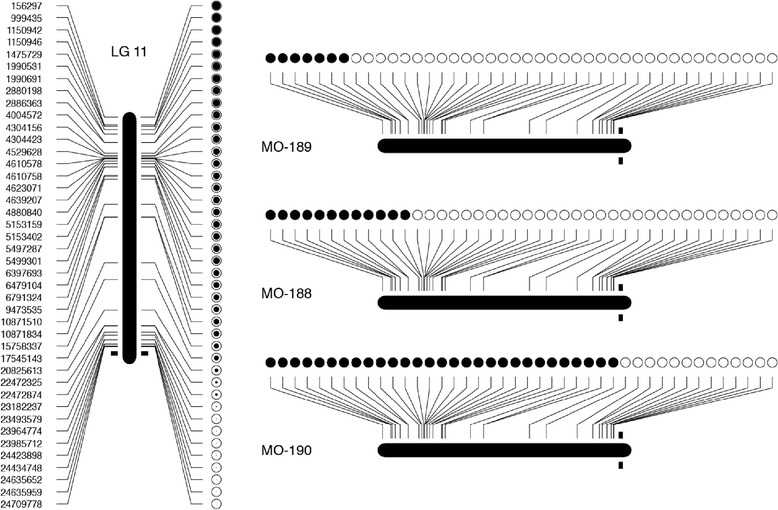
Detailed example of recombination mapping in a single sea bass linkage group (LG 11), illustrating on the left side the computed recombination fraction for 79 progeny. Empty circles represent homozygotes close to the centromere (represented by black boxes either side of the linkage group), and increasingly filled black dots represent higher frequencies of heterozygotes towards the telomeric region. The panel to the right represents randomly chosen individuals from the meiotic gynogenetic family, showing the recombination points in LG 11

Eleven chromosomes (LG 1B, 2, 3, 6, 10, 11, 12, 15, 16, 18–21 and 20) showed single armed (mono-armed) behaviour, with heterozygosity rising from one end of the chromosome to the other reaching up to almost 100%. Figure 4 shows an example of crossover points in a mono-arm chromosome (LG11) in individual progeny, with the overall pattern for LG11. Three chromosomes (LG 4, 19 and 22–25) fitted the mono-armed pattern with the exception of a single outlying marker (i.e. the heterozygosity value for one marker did not fit the overall pattern). Three chromosomes (LG 14, 17 and 24) represented a clear bi-armed pattern (intermediate region with very low heterozygote frequency, rising towards a high frequency at either end). One chromosome (LGX) fitted the bi-armed pattern with the exception of a single outlying marker (i.e. the heterozygosity value for one marker did not fit the overall pattern). Six chromosomes (LG 1A, 5, 7, 8, 9 and 13) did not show a clear pattern of heterozygosity along the chromosome that could enable us to assign an arm structure (mono-armed or bi-armed). This is summarised in Table 1.

To explore this further, we compared the RAD locus positions from the dense linkage map of Palaiokostas et al. [19] with those in the genome assembly. All of the linkage groups of the [19] map contained markers from the corresponding chromosome in the genome assembly, plus additional markers from unassigned (UNK) genome scaffolds. There were no cases where markers were assigned to different chromosomes in the assembly. The correlations for each linkage group are shown in Additional file 8: Table S6. The six LGs which did not show a clear pattern of heterozygosity in the current study were all among the 10 LGs showing the lowest correlation in marker order between the dense linkage map and the physical assembly, suggesting an association between the accuracy of the genome assembly and the clarity of arm structure derived from the present data. Of the 764 ddRADseq markers in the linkage map based on the meiotic gynogenetic family, 63 (8.2%) were also found in the denser RADseq linkage map of [19]. All of these were found in the same linkage groups in both maps, and in the LGs with more than one such marker, the marker order in the present map corresponded to that of the denser map [19].

After removing the six chromosomes that did not show clear heterozygosity patterns (LG 1A, 5, 7, 8, 9 and 13) and the single anomalous markers in three chromosomes (LG 4, 19 and 22–25), the mean recombination frequency per chromosome arm was 0.989 (S.E. 0.123). However, there were instances of multiple crossovers (an average of 11.36% overall) in some chromosome arms (see Additional file 9: Table S7).

## Discussion

The present study constructed the first gene-centromere linkage map (of moderate marker density) for the European seabass, in order to identify markers at the distal end of the chromosomes. Such markers are more informative in discriminating between mitotic and meiotic gynogenetics, due to their higher recombination frequencies. Given the large number of markers showing high frequencies of heterozygotes in the meiotic gynogenetic family (almost half with >67% heterozygotes, including seven with 100% heterozygotes), there would be a vanishingly small probability of mistaking a meiotic gynogenetic for a mitotic gynogenetic using such a marker set. This study also explored a second technical issue in the production of gynogenetic fish, that of potential paternal contribution following UV irradiation of sperm, by analysing large numbers of informative SNP markers (compared to smaller numbers of markers in previous studies on fish species).

The genotyping-by-sequencing approach used in this study (ddRADseq) proved to be very successful for both objectives, and also to be cost-effective for this purpose, generating 804 informative markers for the gene-centromere map and 340 informative markers for assessing potential paternal contribution, from the analysis of a single ddRADseq library (in two sequencing runs). It is feasible to prepare and sequence such a library in one to two weeks for relatively modest cost, and this technique could thus be used routinely in verifying the development of isogenic lines in this and other fish species. RADseq [46] and its derivative ddRADseq [31] have already been used for genetic linkage mapping in model and non-model organisms [47–51], studies on sex determination systems [52, 53] and QTL analysis [54].

A requisite for successful production of uniparental fish is the ability to completely inactivate the genetic material in the irradiated gametes. In this study, 340 male informative SNP markers were identified, none of which were detected in any of the 79 progeny. These markers were located across all 24 linkage groups, confirming a lack of paternal contribution at this level of resolution. It is clear that using this protocol [23] we were able to produce a robust gynogenetic family, suitable for gene-centromere mapping.

A genetic linkage map, comprising 764 SNPs spanning 1251.02 cM with an average marker distance of 1.63 cM, was constructed. Approximately 95% of the female-informative SNPs (764 out of 804) were successfully placed on the linkage map. The genetic linkage map constructed in the present study was shorter than the denser map produced by [19], which had a total length of 4816 cM. The length of *D. labrax* linkage groups in the present study varied from 22.79 cM to 78.04 cM and exhibited a positive correlation, in most cases, with the number of markers mapped per linkage group. Marker-centromere frequencies ranged between 0 and 1 (0 and 50 cM). These results clearly demonstrated that SNP loci produced by ddRAD sequencing were widely distributed in the seabass chromosomes, covering the entire chromosomal regions from proximal (centromeric) to distal (telomeric) regions. Theoretically under the assumption of no interference (with multiple crossover events potentially taking place between non-sister chromatids), the maximum frequency of heterozygotes should be 67% at the telomeres. However out of 804 female heterogametic SNP loci, 395 loci (49.12%) showed heterozygote frequencies above 0.67, indicative of crossover interference in seabass chromosomes. This phenomenon is well documented in the literature for other fish species [4, 8, 43, 55]. Similar proportion of markers (48.1%) with heterozygosity exceeding 0.67 were observed in turbot (*Scophthalmus maximus*) [56]. Twenty-seven of the seabass SNPs showed over 90% heterozygotes in the meiotic gynogenetic family (of which seven showed 100% heterozygotes), suggesting that these could be used in individual SNP assays as a smaller scale assay for discriminating between meiotic and mitotic gynogenetics. At the centromeres of the chromosomes, 68 loci showed less than 10% heterozygotes (of which 16 showed no heterozygotes). We detected at least four chromosomes that appeared to be bi-armed, rather more than the 0–2 biarmed chromosomes detected by karyotypic analysis (reviewed by [45]). The resolution of very small short chromosome arms from basic karyotyping is fairly poor, and it seems likely that applying large numbers of markers in studies such as the present one will result in the detection of more bi-armed chromosomes.

High levels of interference were reported in rainbow trout (*Oncorhynchus mykiss*) [4]. Subsequent literature suggests that high crossover interference is a wide-spread phenomenon in fish and shellfish species [4, 55–58]. The results from the present study in general support this, with an average recombination frequency of around one per chromosome arm (0.98 ± 0.12 (SE), see Additional file 5: Fig. S1). However some multiple crossovers were observed (an average of 11.36%), suggesting that interference is not complete. The high marker density in this study probably helped to detect these events.

It was not possible to construct a genetic linkage map directly from the meiotic gynogenetic genotypic data in this study. It was not entirely clear if this was due to the nature of the data or the fact that linkage mapping softwares were not developed for this type of family. However, after defining linkage groups from the distribution of the markers in the sea bass genome assembly, we were able to order markers within these linkage groups with subsequent analyses, suggesting that this was a successful approach. We suggest that in any future similar studies, it would be better to produce a diploid biparental family as well as a meiotic gynogenetic family from the same parents, then the recombination data could be overlaid onto the linkage map constructed from the biparental sibs, which should contain essentially the same set of markers. This approach was followed to some extent previously in a study on rainbow trout [59] (*n* = 60 in meiotic gynogenetic family; *n* = 60 + 60 in two F1 crosses between two isogenic lines), however the meiotic gynogenetic family was only used for finding intervals where centromeres were located in the duplicated genome of the rainbow trout from a limited number of loci (pers. comm., R.Guyomard). These authors did not describe any attempt to construct a linkage map from the meiotic gynogenetic data.

Isogenic lines are likely to be a valuable resource for research on genetic improvement of complex traits in aquaculture of European seabass, as has already been demonstrated in other fish species, principally the rainbow trout [2]. Androgenesis appears to be an attractive route towards developing isogenic lines, and should lack the complication of spontaneous meiotic gynogenetics. However, the major problems encountered in inducing androgenesis in sea bass using UV irradiation of eggs [21], and indeed the paucity of publications on successful induction of androgenesis in other marine teleosts [2, 60], suggest that mitotic gynogenesis is currently the more likely successful route towards isogenic line development in this species.

## Conclusions

In an effort to define telomeric markers to aid in the reliable production of isogenic lines by differentiating between meiotic and mitotic gynogenesis, we constructed a genetic linkage map and a gene-centromere map from a meiotic gynogenetic family of European seabass. This is the first genetic linkage map based on a meiotic gynogenetic family, although it was not possible to construct this de novo, so the draft genome of the sea bass was used for initial definition of the linkage groups. While there was high congruence between the genetic map from this study and the higher density map of Palaiokostas et al. [19], six linkage groups showed a lack of clarity in arm structure and low correlation in marker order between the dense linkage map of [19] and the genome assembly. This may reflect issues in the accurate assembly of these chromosomes in this first draft sea bass genome (dicLab v1). The data from the two linkage maps could be used in improving the genome assembly and interpreting the genomic data.

In the mapping family analysed, no paternal contribution was detected, validating the protocol used for UV inactivation of parental genome. The large number of telomeric and subtelomeric markers (i.e. those with high percentages of heterozygosity) in the meiotic gynogenetic family suggest that this approach should easily distinguish between meiotic and mitotic gynogenetics thus advancing/supporting future chromosomal set manipulation procedures in this species.

## Additional files

Additional file 1: Table S1. Detailed information for each sample used: Sample ID, origin, UV irradiation and shock parameters, sampling tissue, fertilisation, sampling date and barcodes used per sample. (CSV 19 kb)

Additional file 2: Table S2. Comparison of ddRADseq runs (1st, 2nd and combined 1st + 2nd sequencing runs). (CSV 5 kb)

Additional file 3: Table S3. All SNP markers used: Marker ID, locations of markers on physical map, genetic map corresponding of LGs, distance (cM) and the percentage of heterozygosity ratio. (CSV 27 kb)

Additional file 4: Table S4. Position of microsatellites from previous studies and their informative level. (CSV 1 kb)

Additional file 5: Fig. S1. Physical map of SNP and microsatellite markers in the European seabass genome. (PNG 2440 kb)

Additional file 6: Dataset S1. Marker ID, physical map location, percentage recombination frequency and sequences (FASTA format). (FASTA 132 kb)

Additional file 7: Table S5. Marker-centromere recombination rate (*y*) and map distances of 804 female heterogametic loci examined in meiotic gynogenetic seabass family. (CSV 45 kb)

Additional file 8: Table S6. Correlation of marker order in genome assembly with that of genetic linkage map of Palaiokostas et al. [19]; n refers to number of markers in common between genome assembly and map of [19]. *:LGs with “ambiguous” arm structure based on heterozygosity pattern in the present study (see main text). (CSV 484 bytes)

Additional file 9: Table S7. Crossover points per chromosome arm. (CSV 3 kb)

## Abbreviations

cM: CentiMorgan
ddRADseq: Double-digest Restriction-site Associated DNA sequencing
LG: Linkage Group
LOD: Logarithm of the odds
SNP: Single Nucleotide Polymorphism
